# Post-Transplant Malignancy in Liver Transplantation: A Single Center Experience

**DOI:** 10.1097/MD.0000000000000310

**Published:** 2014-12-02

**Authors:** Chih-Yang Hsiao, Po-Huang Lee, Cheng-Maw Ho, Yao-Ming Wu, Ming-Chih Ho, Rey-Heng Hu

**Affiliations:** From the Department of Surgery (CYH, PHL, CMH, YMW, MCH, RHH) and Graduate Institute of Clinical Medicine, College of Medicine, National Taiwan University, Taipei, Taiwan (PHL, CMH).

## Abstract

We aim to determine the incidence of malignancy after liver transplantation (LT) compared to general population.

The records of patients who received LTs at our center from October 1989 and November 2012 were retrospectively reviewed. The standardized incidence ratio (SIR) of cancer in the patients was compared to general population using the data from the Taiwan Cancer Registry. Survival was estimated using the Kaplan–Meier method.

A total of 444 patients were included. Malignancy was found in 46 (28 de novo and 19 recurrent malignancies) patients (10.4%) with the median follow up of 4.2 ± 4.2 years. The median time of cancer occurrence after transplant was 1.2 ± 1.9 years (range, 0.2–9.1 years). Post-transplant lymphoproliferative disorder was the most frequent de novo malignancy (57.1% [16/28]). The cumulative incidence rates of all malignancies were 5.1%, 10.4%, 12.8%, 15.8%, and 15.8% at 1, 3, 5, 10, and 15 years, respectively. The cumulative incidence rates of de novo malignancies were 3.4%, 5.97%, 7.7%, 10.9%, and 10.9 % at 1, 3, 5, 10, and 15 years. Compared to general population, transplant recipients had significantly higher incidence of all de novo cancers (SIR: 3.26, 95% confidence interval [CI]: 2.17–4.72), hematologic (SIR: 58.4; 95% CI, 33.3–94.8), and bladder (SIR: 10.2, 95% CI: 1.1–36.7) cancers. The estimated mean survivals after transplantation in cancer-free, de novo cancer, and recurrent cancer patients were 17.7 ± 0.5, 11.3 ± 1.2, and 3.6 ± 0.6 years, respectively.

There is a significantly increased risk of malignancies after LT in the Taiwanese population.

## INTRODUCTION

Patient and graft survival after liver transplantation (LT) have progressively improved in recent decades. Post-transplant malignancy, however, remains a leading cause of death and accounts for more than 20% of deaths during long-term follow-up.^1^ The risk of de novo malignancy following LT is significantly higher than that of the general population, with standardized incidence ratios (SIRs) ranging from 2.3 to 4.3.^2–5^ Skin, hematological, and colon cancers are common de novo malignancies after LT.^2–5^ Immunosuppression plays a major role in oncogenesis in the transplant population.^6^ Other risk factors included hepatitis C virus (HCV) infection, smoking, alcoholic cirrhosis, and sun exposure.^7,8^ Understanding the prevalence and risk factors of post-transplant malignancy may help establish screening programs to promote early diagnosis and improve survival of LT recipients.^9^

Previous studies regarding malignancy after transplantation were mainly performed in kidney recipients.^10^ A similar incidence, but different type of de novo malignancies after kidney transplantation between Western and Asian countries has been reported.^11^ Data of LT recipients with post-transplant malignancies from Asia is limited.^10^ Malignancies after solid organ transplantation are generally de novo, except for those after LT because the liver is the only solid organ in which malignancy can be treated by transplantation. Recurrent malignancies are rare after heart, lung, and kidney transplantations because patients with active malignancies are not regarded as good candidates for transplantation of those organs. Patients who undergo LT for liver malignancy sometimes develop cancer recurrence after the transplantation. Compare with other solid organs, both de novo and recurrent malignancy can occur after LT, making this topic complex and important.

The aim of this study was to describe the incidence, cancer types, outcomes, and risk factors of patients who developed a malignancy after LT at a single center.

## MATERIALS AND METHODS

### Patients

The Institutional Review Board of National Taiwan University Hospital, Taipei, Taiwan approved this study (NTUH REC: 2014100006RINA). From October 1989 to November 2012, 444 LTs were performed at National Taiwan University Hospital. All 444 patients were followed until January 2013, and were included in this study. Patient demographic data were retrospectively collected from medical chart review. All patients received regular monthly or bimonthly follow-up at the outpatient clinic after transplantation. Routine blood tests for liver function and tumor markers were checked at each visit, and abdominal sonography was performed every 3 to 6 months. If patients had specific complaints or suspicious lesions, additional imaging studies such as chest radiography or computed tomography (CT) were performed. The diagnosis of malignancy was confirmed by histopathological tissue examination or a typical contrast-enhanced image pattern, such as that seen with hepatocellular carcinoma (HCC). Patients with hepatobiliary cancers such as HCC, cholangiocarcinoma, and hemangioendothelioma were eligible for LT as long as there was no evidence of major vessels invasion or extrahepatic metastasis at the time of transplantation. Patients with HCC were required to meet the Milan criteria (before 2006) or the University of California, San Francisco (UCSF) criteria (since 2006) at the time of LT. De novo malignancy was defined as development of a new cancer after transplantation without a prior history of such cancer. None of the patients was lost to follow-up. The study cohort was compared to the national population using data from the Taiwan Cancer Registry Annual Report published by the Bureau of Health Promotion Department of Health, the Executive Yuan, Taiwan, in 2009.^12^

### Immunosuppression

The immunosuppression protocol after LT consisted of a calcineurin inhibitor (cyclosporine or tacrolimus), mycophenolate mofetil, and steroid therapy. The calcineurin inhibitor (mainly tacrolimus) was given orally after LT beginning the first day postoperatively, and continued with dose adjustments to achieve therapeutic drug levels taking into account renal function. Basiliximab was administered immediately before graft reperfusion, and on postoperative Day 4 as induction. Methylprednisolone given as a 500 mg intravenous bolus immediately before reperfusion of the graft, and then tapered to oral prednisolone over 1 week and subsequently tapered to discontinuation over 6 months.

### Statistical Analysis

Data were expressed as mean ± standard deviation, median (interquartile range [IQR]), or number (percentage) when appropriate. Student's *t*-test, the χ^2^ test, or Fisher's exact test was used for intergroup comparison. Survival curves were estimated using the Kaplan–Meier method, and compared using the log-rank test. Multivariate analysis was performed based on the Cox proportional hazards regression model. A value of *P* < 0.05 was considered significant. All statistical analyses were performed using SPSS 18.0 for Windows (SPSS Inc., Chicago, IL).

## RESULTS

### Patient Demographic Characteristics

A total of 444 LT patients with follow-up of 2281 person-years were included in the study. The median duration of follow-up was 4.26 (IQR: 1.49–8.63) years (mean, 5.15 ± 4.19 years). There were 253 males (57%) and 191 females, and the mean age at transplantation was 38.5 ± 23.2 years (range, 4 months–71 years; median, 49.0 years). Patient demographic data and clinical characteristics are summarized in Table 1. There were 320 adult (≥18 years of age) and 124 pediatric recipients. Of the 444 patients, 327 underwent living donor transplants (73.6%) and 117 underwent cadaveric (26.4%) transplants. The main indications for LT were cirrhosis (33.1%), liver malignancy (26.4%), biliary atresia (18.9%), and fulminant hepatitis (11.9%).

**TABLE 1:**
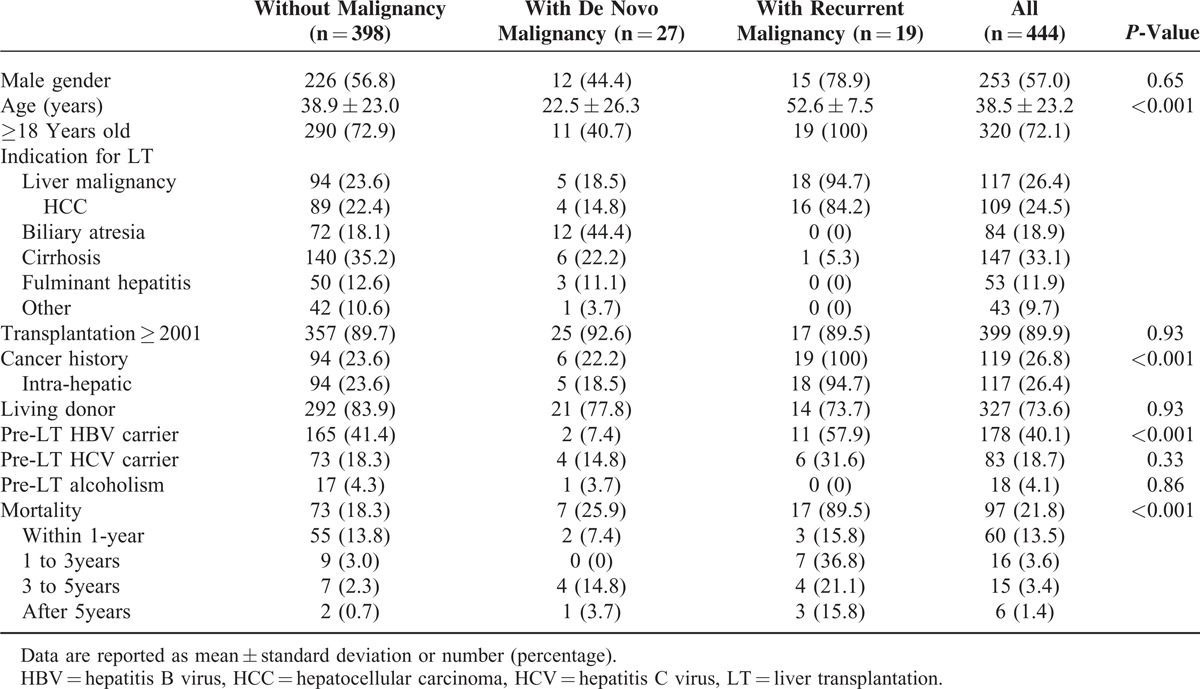
Characteristics of Liver Transplant Recipients With and Without Post-Transplant Malignancies

### Cause of Deaths After LT

Of the 444 patients, 97 (21.8%) died after LT and 27 (6.1%) died within 30 days after transplantation. The main causes of surgical mortality were infection (12/27, 44.4%), graft dysfunction (6/27, 22.2%), and bleeding (4/27, 14.8%). From 31 days to 1 year after transplant, infection was the primary cause of death, which accounted for 20 (62.5%) deaths in 32 patients, followed by 6 (18.8%) deaths due to graft dysfunction and 4 (12.5%) due to malignancies. From 1 to 5 years after transplantation, malignancies accounted for 13 (41.9%) deaths in 31 patients, followed by 10 deaths due to (32.3%) infections and 2 deaths due to (6.5%) chronic rejection. Seven patients died 5 years or more after transplantation, and 3 deaths were due to malignancy.

### Characteristics of Malignancies After LT

Of the 444 transplant recipients, 46 patients developed 47 malignancies, including 28 de novo malignancies among 27 patients and 19 recurrent malignancies among 19 patients. The incidences of overall and de novo cancers were 2017 and 1228 cases per 100,000 person-years, respectively. The clinical data of the 46 patients with post-transplant malignancies are summarized in Table 2. Among the 46 patients, there were 30 adults (65.2%) and 16 pediatric recipients (34.8%), and 11 patients (23.9%) received cadaveric LT and 35 (76.1%) received living donor liver transplantation. The median age at the diagnosis of malignancy was 49.7 (IQR 3.5–59.8) years (mean, 36.8 ± 25.8 years; range, 0.98–66.1 years), and the median interval from LT to the development of malignancy was 14.1 (IQR, 8.2–31.5) months (mean, 22.6 ± 22.5 months; range, 2.7–108 months).

**TABLE 2:**
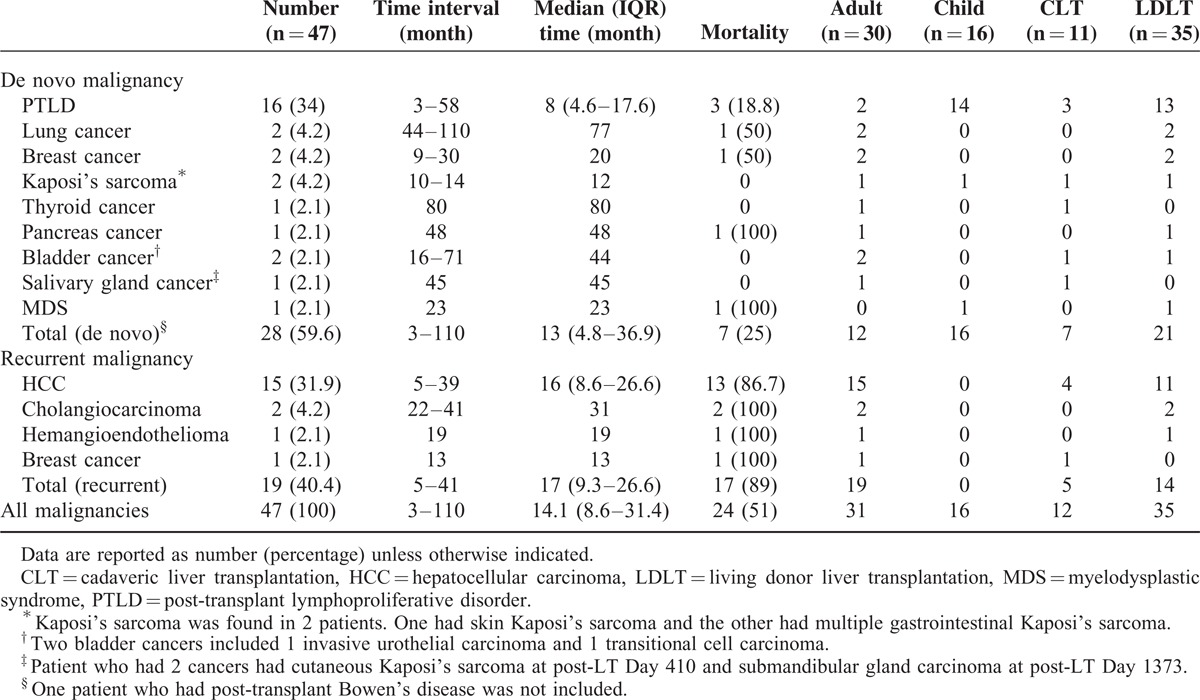
Cancer Type, Malignancy-Free Interval, and Mortality After Liver Transplantation

Recurrent malignancies accounted for 19 of the 47 malignancies, including 15 HCCs (6 recurred in the lung, 5 in the liver, 3 in bone, and 1 in the brain), 2 cholangiocarcinomas (1 recurred in lung and bone and 1 in muscle), 1 hemangioendothelioma that recurred in the liver and bone, and 1 breast cancer that recurred in bone. Seven of the 19 patients (37%) developed recurrence within the first year post-LT, 17 of the 19 patients (89%) developed recurrence within 3 years post-LT, and all recurrences developed within 4 years after LT.

The most common de novo malignancy was post-transplant lymphoproliferative disorder (PTLD), which occurred in 16 patients. Other de novo malignancies included 2 breast cancers, 2 lung cancers, 2 bladder cancers (1 transitional cell carcinoma, and 1 invasive urothelial carcinoma), 1 pancreatic cancer, 1 cutaneous Kaposi's sarcoma, 1 gastrointestinal Kaposi's sarcoma, 1 thyroid papillary cancer, 1 myelodysplastic syndrome, and 1 submandibular gland lymphoepithelial carcinoma. One patient had 2 malignancies. He was a 52-year-old male who underwent cadaveric LT because of recurrent HCC and developed cutaneous Kaposi's sarcoma 13.5 months after transplantation, and developed submandibular gland cancer 45 months after LT; both were de novo. He received sirolimus as the sole immunosuppressant, curative resection of regressed Kaposi's sarcoma, and concurrent chemoradiotherapy for submandibular carcinoma. He had recurrence of the cutaneous Kaposi's sarcoma and subsequently received 3 resections in the following 7 years. Metastatic lymphoepithelial carcinoma in the abdominal lymph nodes was found 4 years after the diagnosis of submandibular gland cancer, and he died of progressive disease.

### Risk of Post-LT Malignancy Compared to the General Taiwan Population

The de novo malignancies after LT and their SIRs compared with the general population in Taiwan are summarized in Table 3. The risk of every de novo malignancy that developed after LT was significantly higher compared with the risk in the general population in Taiwan (SIR = 3.26, 95% confidence interval [CI] 2.17–4.72). SIRs were significantly elevated in 2 specific malignancies, PTLD (SIR = 62.2, 95% CI 35.5–101.0) and bladder cancer (SIR = 10.15, 95% CI 1.14–36.67). The risks of developing other malignancies were also noted to have a tendency to be increased.

**TABLE 3:**
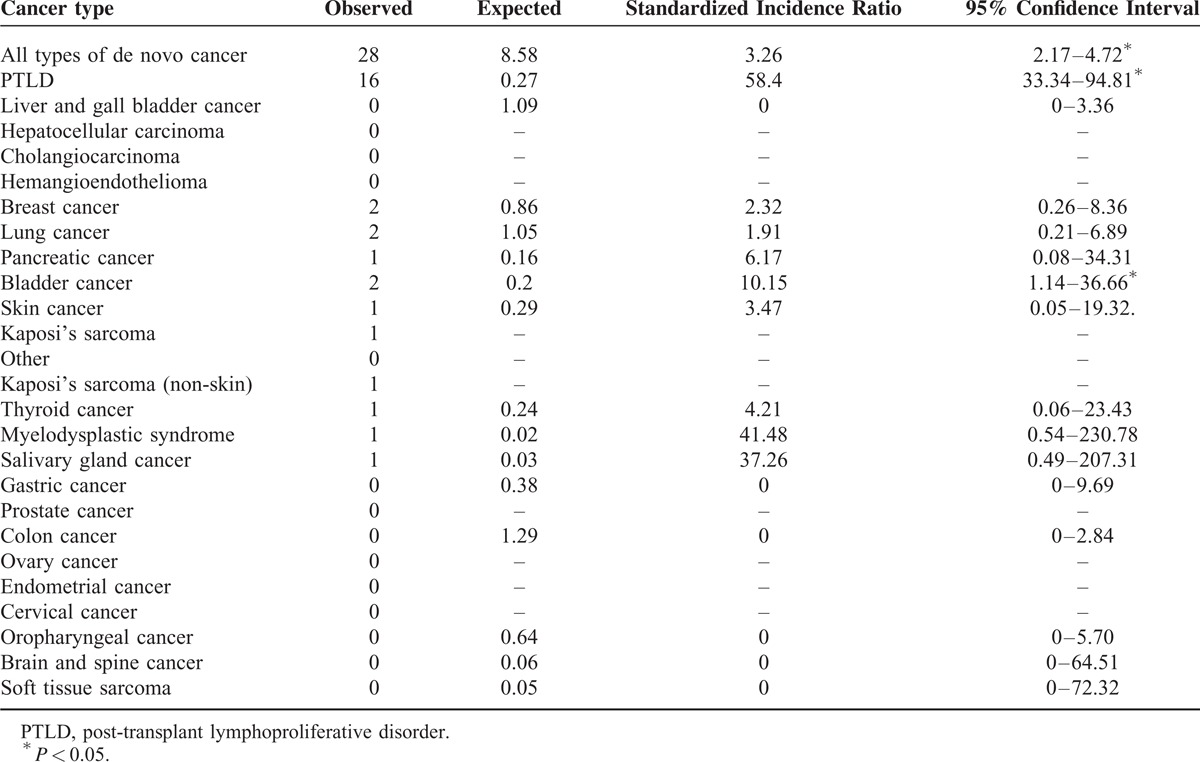
Observed and Expected Occurrence, and Standardized Incidence Ratios of De Novo Malignancies After Liver Transplantation

**FIGURE 1:**
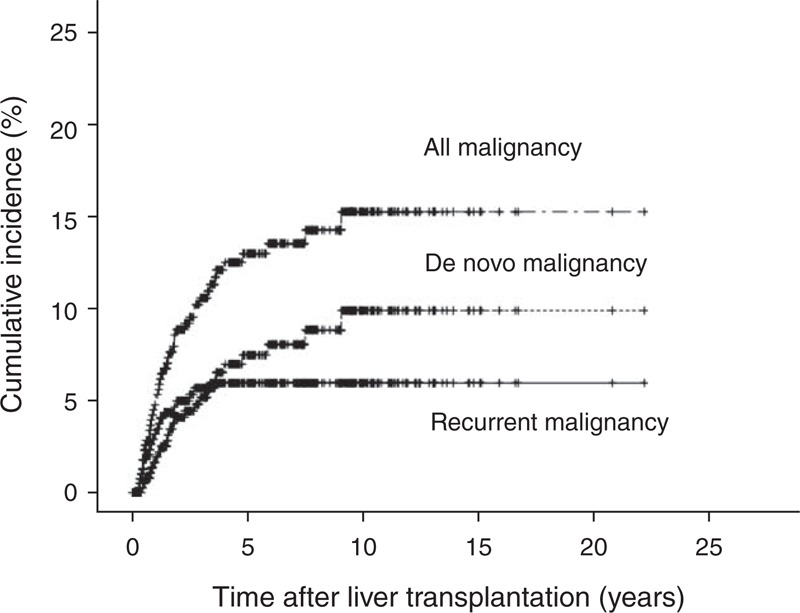
Cumulative incidence of all, de novo, and recurrent malignancies after liver transplantation.

The cumulative incidences of post-transplant malignancies represented by Kaplan–Meier curves are shown in Figure 1. The cumulative incidences of all malignancies at 1, 3, 5, 10, and 15 years after transplantation were 5.1%, 10.4%, 12.8%, 15.8%, and 15.8%, respectively, and for de novo malignancies were 3.4%, 6.0%, 7.7%, 10.9%, and 10.9%, respectively. Of the 46 patients who developed a malignancy after LT, 24 (52.2%) died. The median time from diagnosis of malignancy to death was 10.9 (IQR 3–28) months (mean, 20.7 ± 29.3 months; range, 0.3–130.3 months).

### Impact of Post-LT Malignancy on Survival

**FIGURE 2:**
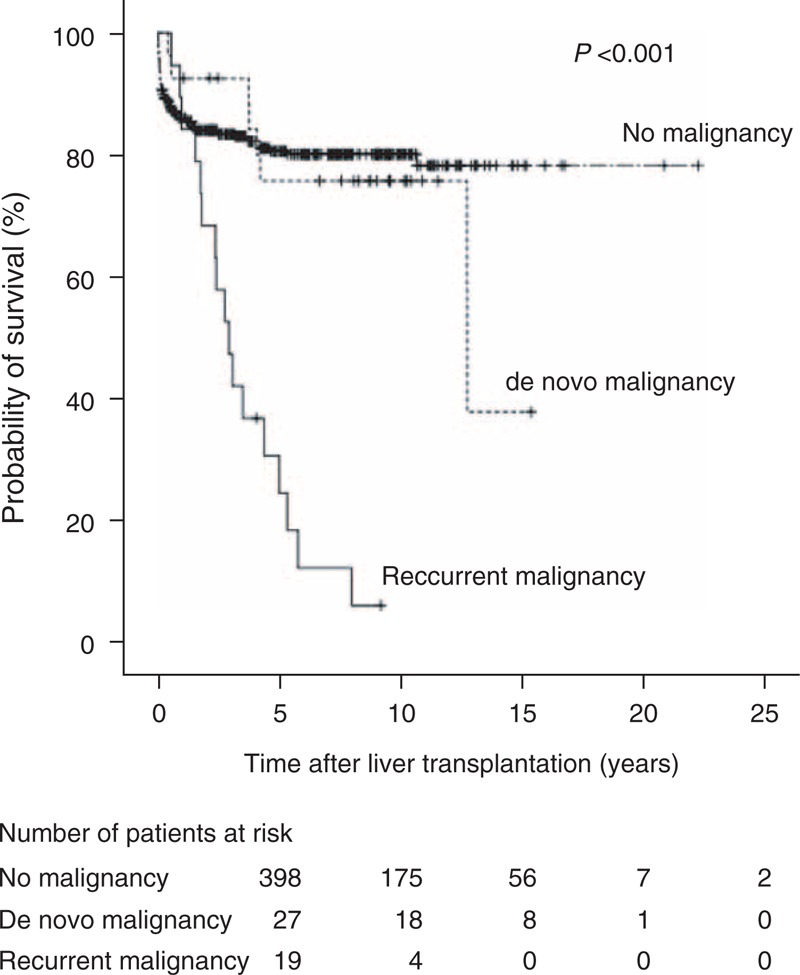
Kaplan–Meier curves for overall survival of liver transplant recipients with de novo and recurrent malignancies and without malignancy.

**FIGURE 3:**
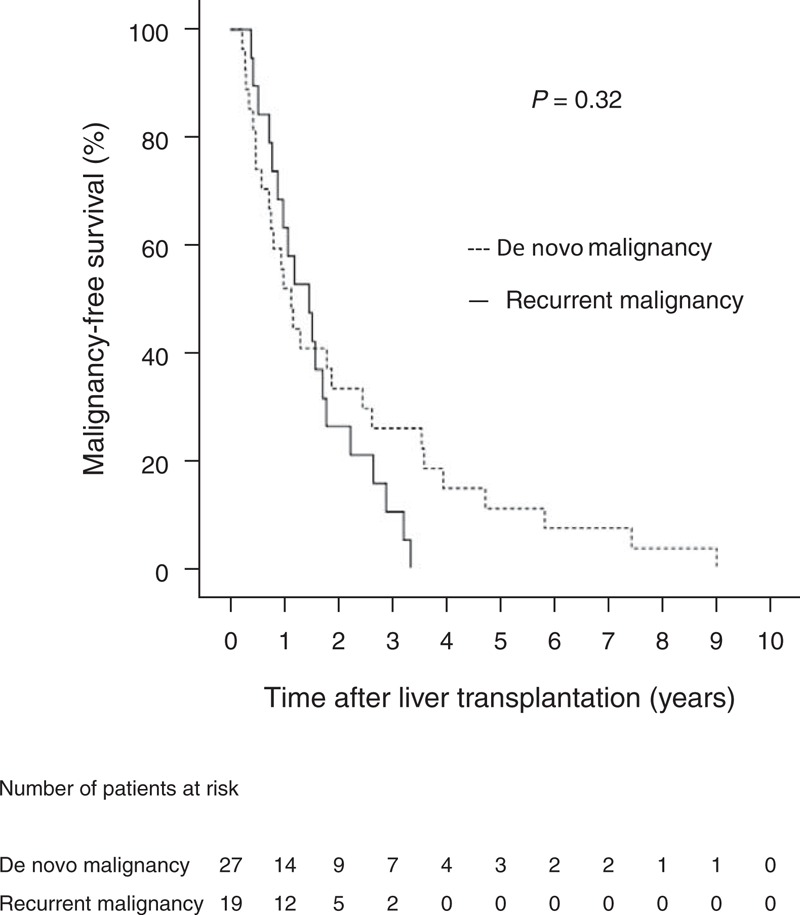
Kaplan–Meier curves for malignancy-free interval in patients who had malignancies after liver transplantations (de novo malignancy vs recurrent malignancy).

The estimated survivals after LT in cancer-free, de novo cancer, and recurrent cancer patients group were 17.7 ± 0.5, 11.3 ± 1.2, and 3.6 ± 0.6 years, respectively. Patients with recurrent malignancy after LT had a significantly higher mortality rate (89.47%, 17/19) compared with patients with de novo malignancies (25.9%, 7/27). There were 6 patients with de novo and 16 patients with recurrent malignancies who died of the malignancy. There were significant differences in survival time between cancer-free, de novo cancer, and recurrent cancer patients (*P* < 0.001), and between de novo and recurrent cancer patients (*P* < 0.001) (Figure 2). Patients without post-LT malignancies had longest survival, followed by those with de novo malignancies, and those with recurrent malignancies. There was no significant difference in the malignancy-free period between patients with de novo and recurrent malignancies (*P* = 0.321) (Figure 3).

### Risk Factors for Post-Transplant Malignancy

Multivariate Cox regression analysis was performed to identify the risk factors of all, de novo, and recurrent malignancies after LT (Table 4). Body weight (hazard ratio [HR] = 0.96, 95% CI 0.93–0.99), cancer history of recipient (HR = 27.79, 95% CI 4.52–170.78), and preoperative fulminant hepatitis as indication for LT (HR = 13.1, 95% CI 1.18–144.67) were associated with all post-transplant malignancy. Body weight (HR = 0.95, 95% CI 0.91–0.99), cancer history of recipient (HR = 25.19, 95% CI 2.18–290.76), pretransplant hepatitis B virus (HBV) status (HR = 0.15, 95% CI 0.02–0.92), preoperative cirrhosis (HR = 16.15, 95% CI 1.55–168.08), and fulminant hepatitis as indication for LT (HR = 53.64, 95% CI 4.61–623.60) were associated with post-transplant de novo malignancy. No factors associated with post-transplant recurrent malignancies were found.

**TABLE 4:**
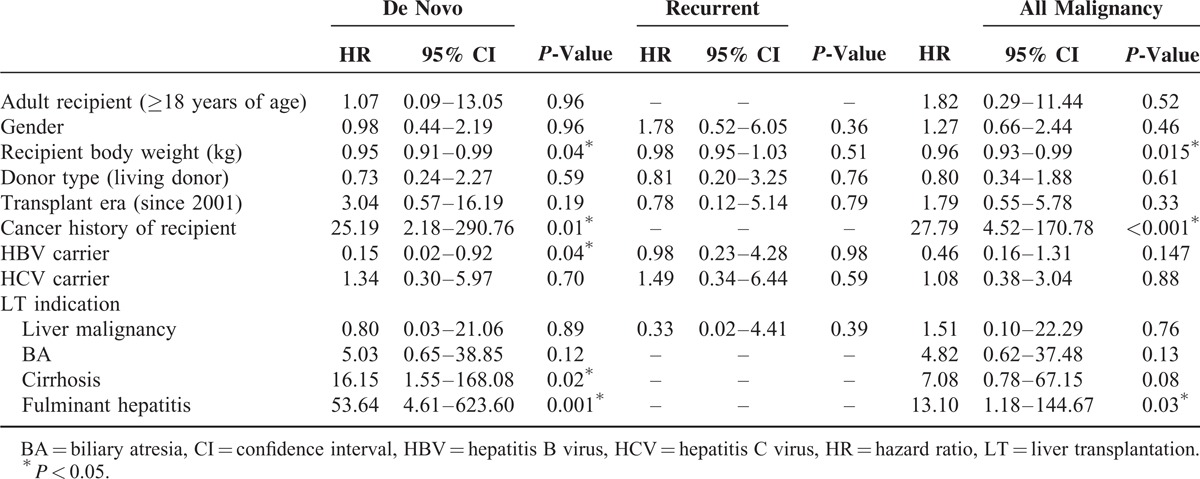
Multivariate Analysis of Risk Factors for Post-Transplant Malignancy

### PTLD After LT

A significantly higher incidence of PTLD was found in the pediatric recipients (14/124, 11.29%) compared with adult recipients (2/320, 0.63%). Among the 14 children, 12 received transplants due to biliary atresia. The majority of patients with PTLD were diagnosed within the first year after transplantation (11/16, 68.75%), and the median time to diagnosis after transplantation was 8.1 (IQR: 4.7–17.9) months. The most frequent site of PTLD was the gastrointestinal tract, including the stomach, duodenum, and ileum, and accounted for 10 of the 16 cases. Other sites involved by PTLD were bone marrow (3 cases), neck (3 cases), liver (2 cases), and breast (1 case). Seven of the 16 patients had more than 1 site involved by PTLD. All patients were treated with anti-CD20 monoclonal antibody and/or chemotherapy, with a survival rate of 81.3% (13/16) and a median followed-up of 7.2 (IQR: 3.1–9) years.

## DISCUSSION

The SIR of de novo malignancy after LT in this study was 3.26 (95% CI, 2.17–4.72) compared with the general population in Taiwan. This overall SIR of de novo malignancy is similar to many previous Western studies that reported a SIR of 2.29–4.3 after LT.^2–5^ Previous studies have reported a SIR of 2.1–4.3 after solid organ transplantation.^2–4,6,13–15^ There are many reasons for the differences between studies. First, there are different exclusion criteria between studies. Studies excluding children^6^ will underestimate cancer incidence because PTLD in children is one of the major malignancies after LT. Studies excluding patients with a cancer history^6,14^ or patients who died within a specific time after transplantation^4^ overlook recurrent malignancies and malignancies that develop within short time after transplantation. Second, there are difference in defining and categorizing malignancies. Some studies included squamous cell carcinoma, melanoma,^3^ or even skin cancer in situ (Bowen's disease)^16^ as skin cancer, but some other studies excluded basal cell carcinoma and squamous cell carcinoma, of the skin,^2^ or even do not include skin cancer.^17^ For example, skin cancer which is often the most frequent de novo malignancy after LT, and accounts for 40% to 50% of all malignancies in transplant recipients in Western studies,^3,14,18–20^ had no significant increase in the current study. Similar results of a low risk of skin cancer in Asian people after heart and kidney transplantation have been reported in several studies.^21–24^ The incidence of skin cancer in the general population is believed to be higher in Western people than in Asian people because of differences in ethnic factors and skin type. Different from Western people, Chinese people do not have an increased risk of developing skin cancer while receiving immunosuppressive treatments after solid organ transplantation. Therefore, when comparing the incidence or relative risk of post-transplant malignancies among different studies, the above factors should be taken into consideration.

This study reported significant higher risks of de novo malignancy with 2 specific cancers, bladder cancer and PTLD. Unlike many post-transplant malignancies, bladder cancer occurs at an increased rate in transplant recipients, but not in HIV/AIDS patients, suggesting that immunosuppression may not be the only risk factor for bladder cancer in patients undergoing transplantation.^6^ PTLD was found in 16 of 444 patients (3.6%), including 14 of 124 (11.3%) pediatric recipients and 2 of 320 (0.6%) adult recipients. It was the most frequent malignancy, and accounted for 57.1% of all de novo malignancies in this study cohort with a very high SIR of 58.4 (95% CI, 33.3–94.8). These data demonstrate a significantly higher risk of PTLD in children than in adults (11.3% vs 0.6%), different from Western studies that showed a similar adult–child ratio.^25^ The median time to develop PTLD in this study was similar to that in another study, which reported median time of 10 months (8.1 months in children).^25^ The most common site of PTLD in this study was the gastrointestinal tract, which is consistent with other reports. The survival rate of PTLD in our study cohort was, however, better than reported in other studies.^25,26^ Targeted monitoring of Epstein–Barr virus (EBV) viral load with preemptive immunosuppression modulation, which is recommended for high-risk pediatric patients for early detection of PTLD, is believed to be the reason for the improved survival in the current study.^27^

We found that most patients with recurrent malignancy developed the recurrence within the first few years post-transplantation. Of the 19 patients with post-LT recurrent malignancy, 7 (37%) developed recurrence within the first year post-LT, 17 (89%) developed recurrence within 3 years post-LT, and all of the recurrences developed within 4 years after LT. Since the immunosuppressive treatment is usually titrated with time after transplantation, these results showed that different intensity of immunosuppression may also impact recurrent malignancy of patients.

In this study, the estimated survival after LT in cancer-free, de novo cancer, and recurrent cancer patients was 17.7 ± 0.5, 11.3 ± 1.2, and 3.6 ± 0.6 years, respectively. Patients with recurrent malignancy had a significantly higher mortality (89.5% vs 25.9%) and lower survival time (11.3 ± 1.2 vs 3.6 ± 0.6 years) than those with de novo malignancies. The impact of malignancy on survival may also be affected by different types of de novo or recurrent cancers. In this study, the major type of recurrent malignancy after LT was HCC and de novo malignancy was PTLD. The difference in survival between the recurrent and de novo groups may be due to the nature of these 2 distinct cancers. Through calculating the tumor doubling time, previous study^28^ reported that recurrent HCC tumors in patients who received LT grew significantly faster than those of patients with HCC tumors who did not receive LT. This may be due to the use of immunosuppressive drugs and the consequent suppression of cell-mediated immunity by impairing natural killer cells, which further suppresses the defense mechanisms against tumor cells and the growth of micrometastasis. These data suggest that patients with a cancer history should be closely monitored for cancer recurrence, especially within the first 3 years post-transplant.

Multivariate Cox regression analysis showed low body weight, cancer history, non-HBV carrier status, and preoperative cirrhosis, or fulminant hepatitis as indications for LT were risk factors for developing de novo malignancy after LT. Low body weight seems especially relevant for pediatric recipients in whom a higher incidence of PTLD was noted. Except for PTLD, low body weight itself may not be a risk factor of de novo malignancy because when PTLD is excluded no more significance was found between body weight and de novo or overall post-transplant malignancy. A cancer history prior to LT was associated with a higher incidence of de novo and overall post-transplant malignancy. This result is similar to that of a previous study, which reported cancer history as a risk factor of de novo malignancy after LT.^29^ Patients with cancer history may have an increased risk of developing de novo malignancies while receiving immunosuppressive treatment after LT. Non-HBV carrier status is associated with an increased risk of de novo malignancy after LT. A possible explanation is the different prevalence of HBV infection between adults and children in the current study due to the successful vaccination policy in Taiwan.^30^ The relatively fewer number of HBV carriers in the de novo malignancy group might simply reflect the fact that children accounted for the majority of the patients in the de novo malignancy group. The fact that preoperative cirrhosis or fulminant hepatitis as indication for LT were risk factors for de novo malignancy after LT in this study requires further investigation. Other potential risk factors, which have been described in previous reports such as different immunosuppression regimes, smoking, alcohol consumption, and sun exposure^7^ were not collected or documented well during our chart review.

This study has some limitations. This study was a single center retrospective analysis, based on the Taiwanese population. The study spanned a long time period, and improvements in surgical and medical expertise and advances in immunosuppression may have influenced the incidence and distribution of malignancies in this study. We did not analyze the effect of different immunosuppression regimes.

## CONCLUSIONS

This study found that LT recipients have a 3-fold greater risk of developing a de novo malignancy compared with the general population, as well as a significantly higher risk of developing PTLD and bladder cancer. The results differ from Western reports with respect to the low incidence of skin cancer after LT. PTLD was the most frequent de novo malignancy, especially among pediatric LT recipients, with relatively good outcomes. The survival of patients with recurrent malignancies was shorter as compared with patients with de novo malignancies after LT.
